# Genomics? That is probably GM! The impact a name can have on the interpretation of a technology

**DOI:** 10.1186/s40504-018-0072-3

**Published:** 2018-04-17

**Authors:** Reginald Boersma, Bart Gremmen

**Affiliations:** Wageningen University, Philosophy Group, Hollandseweg 1, Bode 188, 6706 KN Wageningen, The Netherlands

**Keywords:** Science and technology communication, Prejudice, Public understanding, Expert–lay communication, Knowledge

## Abstract

We investigate how people form attitudes and make decisions without having extensive knowledge about a technology. We argue that it is impossible for people to carefully study all technologies they encounter and that they are forced to use inferences to make decisions. When people are confronted with an intangible abstract technology, the only visible attribute is the name. This name can determine which inferences a person will use. Considering these inferences is important: first, a name will reach consumers before detailed information, if any, will. Second, if detailed information reaches consumers, the hard-to-comprehend information is processed using pre-activated attitudes and beliefs. Using the available literature, we explore the impact a name can have on the interpretation of a technology. We argue that science communication can benefit from trying to develop a name for a technology that activates proper beliefs to guide non-experts to a more meaningful understanding of it.

## Introduction

People often come in contact with a new technology in situations where they have hardly any relevant knowledge or information. In situations like these, a name can be very influential. For example, imagine yourself going shopping for your favourite food. When you arrive at the place where your favourite dish is sold, you suddenly notice that, besides the regular food you are used to buying, there is a new option. This option is produced using a technology called *next gen-radiation*. How will you give meaning to this new technology, in the middle of the supermarket, without books, internet or experts on the matter to explain to you what it is? What information will you use? Without anything else, you only have the name to go on.

*Genetic manipulation* is a good example of a technology name that influences perceptions. To manipulate means to control, and so from a technological perspective the term gives a good representation of what the technology entails from an expert perspective. Unfortunately, when the term reached the public, people became suspicious of it, and so the term itself contributed to the negative perceptions towards the technology because of the negative connotation of manipulation (Hansen 2010). In an attempt to reverse the damage, the name genetic manipulation was changed to *genetic modification* (Hansen 2010). This shows that the term used is regarded as an important factor in the interpretation and acceptance of the technology.

Currently, a new approach to genetics is being developed under the name *genomics*. The term genomics can refer both to the science of genomics, which is the study of the genome, and to the application of the science, for example genomics-assisted breeding. Applied in plant breeding, genomics-assisted breeding can be used to create new crops more efficiently by using knowledge on the relation between genomes and traits. Genomics is similar to traditional breeding in the sense that crops are combined through sexual reproduction to create a new variety. Genomics enables plant breeders to check whether the genome of the new variety contains the parts that can cause the desired traits without having to wait until the parts come to expression, thus accelerating the process significantly. It differs from genetic manipulation, where traits are introduced artificially. Therefore, genomics can serve as an alternative to this controversial technology and is even being promoted as such by Greenpeace (van Aken 2009), one of the major opponents of genetic manipulation.

Unfortunately, even though genomics circumvents the main objection against genetic manipulation, people react very similarly to the technology (van Dam and de Vriend 2002). It appears that the similarity of the names is affecting the perception of genomics negatively. In a Dutch study on perceptions about genomics, the authors conclude that the respondents do not know what genomics is and that they have difficulty understanding all the information they receive (van Dam and de Vriend 2002). Nevertheless, they answer the question posed to them as if they do understand. The authors speculate that the respondents are actually using knowledge about genetic manipulation to formulate answers. In fact, they observe that different respondents automatically use the term genetic manipulation, especially when genomics is mentioned in the context of plant breeding.

The essence of the problem in the case of both genetic manipulation and genomics is that people who do not know much about the related scientific or technological concepts react in a way that is unexpected by experts, i.e., as if they do have knowledge about the concepts. When conflicts arise, scientists tend to believe that these unexpected reactions are caused by a lack of knowledge on the part of the public and that the conflicts can be countered through education (Ahteensuu 2012). In situations where conflict has appeared, the public have been called passive (see Bucchi 2008) or indifferent (Bodmer 1985) for not informing themselves about the relevant technology. We argue that this does not do justice to the public. People simply do not have the time to study all the innovations they may encounter, as we argue in more detail later.

In the current article, we investigate how people give meaning to an unfamiliar technology about which they have no knowledge other than the name, and the way this process is influenced by the name of the technology. We argue that different names can cause different inferences to be created, influencing the way people interpret a technology. Whereas one name might assist useful interpretation, another name might cause interpretations using concepts that are irrelevant. Although there will be some attention to the effects of a name on the possible processing of information when it is available, the main focus of this article will be on responses triggered by just the name of a technology. As such, we depart from the dominant approach in science communication where the effects of provided information are investigated (Bos et al. 2009) and influences of cognitive mechanisms such as framing, (de)coding and the elaboration likelihood model on the interpretation of information is investigated.

We want to stress that it is not the aim of this article to promote merely choosing a nicer sounding name. The aim is to investigate the development of knowledge and its effects on attitudes and the way this is influenced by a name, so that we can have a deeper understanding of the process – an understanding which can help narrow the communication gap between experts and the public. We conclude that science communication can benefit from an approach in which the development of a name becomes a process of co-creation between science and society. We begin by presenting a reflection on science communication from a cognitive perspective, to highlight where we believe a lacuna exists.

## History of understanding and attitudes from a cognitive perspective

In 1985, the report entitled *The Public Understanding of Science* was published (Bodmer 1985). The report was initiated because of the increasing public scepticism about science and technology (Gregory and Lock 2008). According to the authors, the negative attitudes towards science and technology were the result of a lack of understanding. Providing people with knowledge through education would solve the problem.

The idea that education solves negative attitudes is now largely rejected in the field of science communication (Ahteensuu 2012). From the current perspective, we identify two groups of issues with the educational model. The first group relates to the role of information in decision making. The second group relates to the political motives behind the report. Both groups have led to developments which still influence the research agenda today and have caused and maintained a void in studying public understanding that we are now trying to fill.

With respect to the first group of issues, the model reasons from the classical economic ‘rational man’ perspective, in which it was presumed that people carefully use all information available to them. A problem with this perspective is that it is not equipped to explain much of human behaviour, and this inspired the development of new insights. In communication, dual process theories on attitude formation (Chaiken 1980; Petty and Cacioppo 1986) have been developed to explain behaviours that could not be explained by the rational man paradigm. According to these models, there are two ways to process information. The first is a systematic way, where all information is elaborated on carefully and which fits the rational man paradigm. The other is a heuristic way, where information is processed quickly and without too much effort. People use the heuristic way for different reasons, among other things because they are not able to devote enough attention to the information – a common event in noisy daily life.

Dual process theories make an important contribution to understanding human decision making. However, they are still about processing externally provided information (Bargh 2002). Much of the attitude formation that takes place in daily life happens without the processing of externally provided information, either because the information is not available on the spot or because daily life offers too much distraction for a person to pay any attention. In conclusion, dual process theories do not account for all possible ways of evaluating. They cannot be applied to explaining attitude formation without external information.

The idea that people form their behaviours without information was successfully advocated by Simon (1979) when he introduced the notion of bounded rationality. According to Simon, when they have to make a decision in daily life, people do not search extensively for all available information. Rather, they use the information currently available to them to make the decision. Whereas dual process theories are about information processing, bounded rationality is about information searching.

With respect to the second set of issues, the political issues, it can be argued that the educational model has given rise to a type of reasoning by technical experts that has led to more conflict with the public. According to Bucchi (2008), members of the public have been called ignorant and lazy for not getting the knowledge that would make them change their opinion, in an attempt to disqualify them from the debate.

Unfortunately, excluding the public because they are ignorant blocks the development of insights into the role that ignorance plays in decision making and attitude formation. From the example where respondents were asked to react to the relatively unfamiliar concept of genomics (van Dam and de Vriend 2002), we can learn that people often do decide and evaluate without knowledge, being ignorant. Within the interaction between the researcher asking about genomics and the respondents, there was no information on genomics available for processing, nor was there familiarity with the concept. Nevertheless, people reacted to the questions. These kinds of behaviours take place in an absence of understanding (or an absence of information, other than the name, to be processed to reach understanding).

Although people are often forced to make decisions without, or before they have, any knowledge about a technology, most research investigates people’s reaction to information about a technology rather than tests behaviours without knowledge. A notable exception is provided in a study of the *ecogenomics* construct (Bos et al. 2009). The goal of the study was to ascertain the amount of information people were planning to try to obtain. Although the respondents reported that they planned to search for information, the amount of this information was very little and far from enough to reach a deeper understanding.

In defence of the Bodmer report, it should be noted that the report argues that public misunderstanding or ignorance can lead to issues in daily life. Even though this may be motivated by political goals, the authors do have a point when they argue that little or no knowledge can lead to misunderstanding. Many technical experts still adhere to the educational model (Ahteensuu 2012), and a possible contributory factor is that they notice that people really do not understand. Given the results in Bos et al.’s (2009) study, it actually would be surprising if the respondents were able to form a proper understanding with the small amount of planned information search.

The respondents’ behaviour becomes understandable when we place it in the greater context of daily life. If people always searched for enough information to reach understanding when they encounter new concepts, they would spend all their time being educated. People have no choice but to act on little information. Rather than try to correct people’s knowledge level, an attempt can be made to account for the way people form their daily understanding. If the process is understood, people could be influenced not to draw inappropriate conclusions and be steered to more appropriate impressions.

A good example of how this might work is provided by the effects of the expressions *global warming* and *climate change*. Some technical experts use the term climate change because it is more appropriate from a technical perspective (Conway 2008). However, with the aim of changing people’s behaviour in daily life, the term global warming might be more efficient. Research has shown that people link global warming more to melting icebergs and glaciers, and the melting is more often believed to be the result of human behaviour (Whitmarsh 2009). Without people fully understanding the complexities of the global climate, global warming triggers the right ideas.

We argue that a better understanding of attitude formation and decision making without information can lead to a more effective way of communicating. Instead of trying to educate the public, technical experts should recognize that daily understanding is a different form of understanding. By explaining how public understanding differs from expert understanding, certain interpretations can be prevented and other interpretations can be enhanced by steering people in the right direction. To be able to do this, we have to understand the differences in the way people and technical experts organize their knowledge, and the way a name representing a technology activates knowledge.

## Understanding new concepts

To understand how human decision making functions, we have to understand how knowledge is organized. A theory that describes this is categorization theory. According to categorization theory, concepts are organized in categories. For example, we have a category for cars, doors and cats, respectively. A category is structured based on the common features of its members (Rosch 1978), and therefore a category contains concepts that are similar in some ways (Loken et al. 2008). Schemata are interconnected categories. A schema of categories is a taxonomic description of the way someone’s knowledge is organized. Categories enable us to use our knowledge efficiently. For example, instead of remembering for each and every cat that it has four paws and a tail, it is enough to remember that, in principle, cats have four paws and a tail.

The usage of categories can in addition help us to understand new unfamiliar concepts (Gregan-Paxton and John 1997; Loken et al. 2008). The notion is that, when people are confronted with new concepts with which they are completely unfamiliar, they study the concept and try to match it with similar concepts they already know. This process is called categorization, which can be defined as placing a new concept in an existing category. Through this act, knowledge about the known category members can be used to interpret the unfamiliar concept.

With categorization, people learn about new concepts through an internal transfer of knowledge. Knowledge about known concepts is transferred to the new concepts. In this respect, it can be considered as an alternative to education, where information is provided externally. When confronted with new concepts, people will first try internal transfer to understand the new concepts (Michaut 2014). The process enables people to quickly form an understanding without the use of any external resources, and therefore with no information search.

Related to the transference of knowledge is the process of attitude extension (Muthukrishnan and Weitz 1991). When attitude extension takes places, the existing attitudes of the known category members are extended to the new concept. In a way, this process serves as a shortcut to forming attitudes without getting knowledge or education. Because of attitude extension, people can project their existing attitudes about familiar concepts onto concepts about which they have no knowledge. It enables them to make quick decisions without knowledge and carry on with their everyday tasks.

The process of attitude extension provides an explanation about where initial attitudes towards science and technology originate. For example, it has been found that a lack of knowledge does not block the formation of attitudes towards genetic modification (Frewer et al. 1994). Attitude extension can account for this process. Attitudes from other, known agricultural technologies, or even other acts of manipulation, can be used to form an attitude about the new technology. Instead of evaluating the new technology using its own attributes, attitudes are copied from other concepts.

A proper way to describe these attitudes is prejudice. Nowadays, the term *prejudice* is virtually exclusively linked to social prejudice; however, prejudice can also relate to concepts. When social prejudice occurs, members of a social category are judged on the attitudes relating to the category to which they belong. When genomics is judged by applying attitudes about genetic manipulation because they are believed to belong to a shared conceptual category, conceptual prejudice occurs. One problem with prejudice is that it is very hard to eradicate. An important reason is that it steers interpretations of new information towards a person’s existing preconceptions.

To prevent inappropriate attitudes from being copied and related prejudice from being formed, the challenge is to anticipate which attitudes will be used for the process. To achieve this, a focus on the activation of categories that are linked to the attitudes is needed, and a focus on how the public’s category structures are different from those of technical experts. It is necessary to understand the difference between technical experts and the public because the difference in structures may lead to unexpected activation of categories and related attitudes on both sides in communication.

Categories can be divided into subcategories containing more detailed information or combined into super categories containing more abstract information (Rosch 1978). In addition, categories can be linked to other categories or concepts. As already discussed, schemata are the resulting networks of categories. For many of these categories or schemata, we have a name or term to represent them (Rosch 1978). When communicating with others, we try to activate these schemata in the mind of the receiver by using the name or term. What the name activates depends to a very large extent on the knowledge present in the receiver. To illustrate, the term *gen* will not mean anything to someone who has absolutely no knowledge about it. On the other hand, it might activate knowledge relating to genetic manipulation for someone who has encountered the term in public debates on genetic manipulation. For an expert, it could mean activation of an extensive schema about heredity. For this expert, genetic manipulation might not be activated directly, because it is regarded by this person as a separate construct. Clearly, expertise is an important factor which determines the sources of knowledge that are activated. To investigate this more precisely, we turn to the topic of how schemata are influenced by expertise.

## Expertise

Expertise is regarded as an important factor in categorization (Alba and Hutchinson 1987). As already stated, different categories can be linked to one another directly or through super categories, and can be divided into subcategories. Compared to non-experts, experts have more elaborate and flexible cognitive structures (Alba and Hutchinson 1987). Therefore, the categorization process can differ greatly between experts and people with less knowledge. To understand how these differences may act out, we discuss the main differences.

Compared to non-experts, experts know more attributes (Alba and Hutchinson 1987). This enables an expert to create a greater number of subcategories than non-experts. Whereas experts and non-experts might both see a laptop when confronted with a MacBook, only the expert will know the attributes that set the MacBook apart from prototypical laptops. In essence, the expert has access to a subcategory that contains extra information. Because they know more, experts can categorize not only more accurately, but also more appropriately. For example, a non-expert might inappropriately categorize a computer screen in the category *televisions*. To understand the difference between a computer screen and a television, a person has to know what a tuner is and use the invisible attribute to divide the concepts into different groups.

Within categorization theory, a differentiation exists between natural categories and abstract categories. Natural categories are categories that people form automatically (Rosch 1978). Abstract categories are transferred through education (Alba and Hutchinson 1987). A good example might be the category *night shade*, to which both the tomato and the potato belong. It requires a certain amount of education to know the category night shade, and why its members are combined the way they are. A non-expert might group a tomato with oranges (in a category called fruit) instead of with potatoes, on the basis of everyday experience.

In addition, there are ad hoc categories (Barsalou 1985). These are created with a particular goal in mind and are formed by focusing on functions or attributes that are relevant within a particular context or with a particular goal in mind. Concepts from different categories that share relevant attributes might end up in an ad hoc category that revolves around these attributes (Barsalou 1985). For example, in certain cases, petrol can remove paint. It shares this attribute with specialized paint removers. The two concepts might end up together in a category constructed with the goal of finding something to remove paint.

Whereas concepts have only one place in the natural categorization classification, they can be simultaneously part of a number of ad hoc categories. For example, although petrol will be by default a member of the category *fuel*, it can end up in several ad hoc categories because of the different attributes of the concept. Another important thing to note is that ad hoc categories can turn into more permanent goal-related categories when they are used more often (Barsalou 1991). Goal-related categories have a similar influence in the processing of information as natural classification structures (Barsalou 1991).

To summarize, experts have both more elaborate structures and a greater number of alternatives to natural categories than non-experts; this enables them to comprehend similarities between different concepts that to non-experts appear unrelated. In consumer behaviour, it has been found that experts tend to use functional attributes for understanding (Gregan-Paxton and John 1997), whereas non-experts use more superficial cues like shape or appearance. Experts can process information about attributes and relate them to other concepts using commonalities between the concepts. In contrast to experts, not having any knowledge about the attributes that can be used to connect the concepts, non-experts are not able to do this.

If a non-expert will have a harder time making proper connections for categorization, falsifying a categorization will be equally or even more difficult. Imagine a person who does not know the technical and functional aspects of a CD. When this person encounters a DVD for the first time, there is a very good chance that the DVD will be categorized with CDs because they are similar in appearance. When a person knows only the superficial features, it is impossible to realize that the categorization is incorrect from a functional perspective. Only knowledge about their respective functional attributes will give the ability to separate the two concepts.

These differences in cognitive structures cause experts to understand more precisely the way new concepts relate to known concepts. An expert with an elaborate understanding of genetic modification, first of all, knows the attributes of the concepts involved. The expert is familiar with attributes like DNA, alleles and genes, and understands how they are related. In addition, the person can separate different approaches such as genetics and genomics. The expert will have the ability to understand that the technology is a new form of reproduction. This realization will enable the expert to position it vis-à-vis other types of reproduction, like sexual reproduction, which in turn is linked to traditional breeding. The reality of genomics-assisted breeding can only be understood using different kinds of schemata.

Whereas experts use functional attributes for understanding, non-experts who do not have the appropriate knowledge about attributes use superficial features for categorization and rely on similarity (Gregan-Paxton and John 1997). Although experts might need the appearance to sense the object, it is the functional attributes that will be used for categorization. For example, an expert will see the same ignition key of a car as the non-expert but consequently know that it will turn the engine on. Therefore, using the functional attribute, the expert might categorize the concept with switches. A non-expert might look for an object that is similar in appearance, for example a lock, in an attempt to understand the new concept.

An important superficial feature of a technology is the name given to it. When a name is given to a technology, any name can be given. However, when a name is chosen to have meaning, the effect of a name on categorization can be similar to that of other superficial features. A meaningful name acts as a conceptual label (Gregan-Paxton et al. 2005). For example, the function of the attribute *door handle* is captured in the name, but will only be noticed by English-speaking people who, in addition, already know about doors and the way they function. So, for people with related knowledge, the name can be a guide to the functional aspects of the attribute (see Rosch 1978). Without the related knowledge, it can guide people to concepts that are similar in name. When a person is confronted with the, for this person, meaningless term genomics, the nearest concept to use to give meaning is genetic manipulation. Whereas tangible concepts can also steer interpretation by their shape (a door handle ‘invites’ its use), the only detectable feature of an invisible technology is the name. People prefer to give meaning using appearance rather than the conceptual label (Gregan-Paxton et al. 2005), but this is impossible when dealing with abstract technologies. Therefore, a name can be very influential in what people believe a technology to be and in the attitudes that are activated. With this in mind, we can answer the question about the way people give meaning to a technology about which they know nothing.

## The effects of a name

Imagine yourself back in the supermarket confronted with the new technology, next gen-radiation. The question is what knowledge you will use to explain to yourself what the technology is. Will the gen part of the name cause you to believe that it has something to do with genetic manipulation, or that it is the next generation of radiation? And what does the term *radiation* remind you of, sunlight, radio waves or ionizing nuclear radiation?

When people try to give meaning to a concept which is communicated by its name, they only have the name to process. The name can determine categorization. By calling a new technology genomics, the name activates meaningful categories for experts. They are given a label that they can use to position the technology vis-à-vis other related technologies, and related knowledge is activated. However, to be able to do this, they need knowledge of these technologies. For non-experts, on the other hand, the term is relatively meaningless and does not guide to a proper categorization. For them, the only effect will be that the genomics concept sounds related to the genetic modification concept. From a learning perspective, the name is unintentionally activating genetic modification as the only available association.

A name can activate knowledge or attitudes other than those intended. Although experts might argue that these are not the right knowledge or attitudes, this knowledge and these attitudes will not be without consequences. People can still make decisions about buying products or supporting a technology using their activated knowledge and attitudes, even if these are linked to irrelevant concepts.

Scientific notions and technologies that are distinctively different according to experts might therefore be experienced as similar by non-experts. This can cause the public to react in a way that is not expected by experts. Attitude extension from genetic manipulation to genomics leads to a negative reaction that might be surprising for experts. Whereas experts feel that they are doing something fundamentally different, or even trying to find a solution for the objections against genetic manipulation by pursuing an alternative which provides similar advantages, the public experience the two as the same thing because they lack the knowledge about the attributes that set the two apart.

Using inappropriate knowledge does not necessarily mean that people will quickly become stuck during processing and understand what is wrong even when additional information is provided. Even though people have activated inappropriate knowledge, the processing of the information can go a long way without the error being noticed. A nice illustration of this is a journalist who reported on the preparation to form the Dutch genomics centre. In his newspaper article, he noted – presumably after having studied the subject – that the centre for genomics was investigating ‘… genetic modification of both the tomato and the potato…’ (Janssen 2002: 10). In the case of genomics and genetic modification, both technologies claim to produce more crops, reduce pesticide use, provide opportunities to produce food in harsh conditions, etc. When more affective attitudes, rather than knowledge, are used to make decisions, it is even harder to notice that they are based on inappropriate information.

An important contributor to not realizing the differences between a new and a familiar concept is selective attention. A name acts as a label guiding people to a particular schema, which in turn acts as a frame when processing information (Ferguson and Bargh 2004; Herr 1986; Higgins 1996). Once this schema becomes activated, people pay more attention to the attributes that are part of the schema, disregarding those that are not (Rajagopal and Burnkrant 2009), which also determines what people will remember (Sedikides and Skowronski 1991). Ironically, these might be the distinguishing attributes that define the newness of the concept. Therefore, even in the few cases where members of the public receive education, the effect of the name can influence the development of the knowledge. In particular, when a new concept is placed close to a related and very similar concept, for example by name, there is an increased chance that people will not notice the difference (Gregan-Paxton and John 1997). Instead of building a new schema, all new information might be connected to the one that has been activated. In the case of genomics, very little applies to genetic manipulation that does not also apply to genomics, and vice versa.

Because experts rely more on functional attributes when they give meaning to a new concept, they more quickly realize the uniqueness of a new concept. For example, genomics can be understood by noticing that the technology shares attributes with both genetic manipulation and traditional breeding. To be able to position the technology within existing knowledge, an expert will create a new schema. Non-experts do not combine knowledge from different sources using attributes the way experts do. Therefore, they do not notice the differences between the new concept and the concept used to give meaning, and consequently do not notice that a single known concept is not enough to understand the new concept. The result is that non-experts learn from single examples (Gregan-Paxton 2001), leading to exemplar learning (Barsalou 1991) and single category beliefs (Rajagopal and Burnkrant 2009). The meaning given to the new concept is basically a copy of what is already known.

To be able to understand what makes genomics a unique concept, a person has to use attributes from different sources. Because of their extensive networks and ability to use attributes to create connections between schemata, experts are able to combine these sources. For non-experts, the problem is that these different sources of relevant knowledge and attitudes are disconnected. A way to combine this knowledge from different sources is described in an approach called learning by analogy (Gentner 1983; Gregan-Paxton and John 1997), which we discuss next. This approach argues that consumer learning can be guided by activating categories that might not be appropriate for categorization but nevertheless contain information that can be useful for understanding.

## Learning by analogy

Learning by analogy argues that, to understand new concepts, we often need more knowledge than is stored in the category used for categorization (Gregan-Paxton and John 1997). Many new concepts that we encounter, especially concepts relating to advanced technologies, can only be fully understood by using knowledge from different categories. For example, smartphones might be categorized in the category *phone*. However, trying to understand a smartphone by categorizing it as a phone will only activate a subset of relevant knowledge and attitudes. Nowadays, smartphones are best understood as being very small portable computers that, in addition to making calls, can run software and be used to connect to the internet. Activating knowledge and attitudes about computers when a person is trying to understand a smartphone might result in a very different understanding and evaluation.

Essentially, learning by analogy proposes a mechanism to circumvent the limitation of learning using single category inferences. The way to achieve this is to single out attributes that are important to understand the concept and to connect these with attributes of known concepts from different categories. According to the theory, communicators should try to achieve understanding by explaining in what ways a new concept relates to others concepts. This can be achieved by illustrating that a smartphone is like a phone, you can make calls, and it is like a computer, you can run software.

Although leaning by analogy can be used to explain new concepts efficiently, it also provides valuable information about situations in which an explanation cannot be provided and where people are forced to act on a name alone. Basically, learning by analogy focuses on unlocking and activating different knowledge than that which might be activated by categorization to enable understanding. Learning by analogy shares with goal-directed categories the idea that there are alternative structures to natural categories to organize knowledge.

When experts search for a name to describe their new technology, they tend to use a taxonomic classification that emphasizes its position relative to other technologies. The name genomics is no exception. It illustrates the relation to other gene technologies. On the other hand, the main attribute of genomics, applied to plant breeding, is that reproduction is not artificial but natural. Genomics shares this attribute with other ways of breeding, for example traditional breeding where crops are produced using natural sexual reproduction. Choosing a name that emphasizes this important attribute, which distinguishes it from other gene technology, provides an alternative that might be more meaningful to non-experts. To exemplify (without suggesting a change of name), genomics could have been called natural crossing instead, to promote the main attribute differentiating it from genetic manipulation.

Whereas learning by analogy emphasizes the notion that there are alternatives to learning through categorization, we emphasize that there is an alternative to naming a new technology from the perspective of category relations. In situations where people do not have schemata based on relations between abstract concepts, a name that stresses an important attribute might contain more information than a name that illustrates the position of a new technology vis-à-vis related technologies.

The meaning of a scientific name can only be clarified on the basis of a dialogue with a broad range of societal actors. Experts have to focus more on the social, cultural and moral (re)presentation of scientific knowledge and the social structure of technology (Hamlett 2003; Decker and Ladikas 2004; Sarewitz 2004). By acknowledging these, experts and social actors can together co-create a name which is both meaningful to the public through the associations. This also provides the opportunity go beyond the associations being meaningful from a theoretical scientific point of view, but to include more normative or moral associations as well In her comparative political study of biotechnology in the United Kingdom, Europe and North America, Jasanoff (2005) showed, that where public involvement is insufficiently available formally, it will occur informally, through public protest; market choices, such as consumer rejection of genetically modified foods; or new political structures, such as environmental movements. By also including normative or moral associations, the risk escalating mistrust as a result of believing a name is misleading or unjust marketing can be diminished.

## Discussion and conclusion

Nowadays, we are continuously confronted with new scientific and technological developments. It is impossible to get to know all of them. The vast majority of technologies will reach people before they become educated about them, if they ever will. That does not mean that people do not respond when they are confronted with concepts with which they are unfamiliar. By using knowledge and attitudes about what they believe to be similar concepts, people nevertheless judge and make decisions.

It is important to realize that, in situations like these, people do not just act at random or irrationally, even though it might appear this way from the perspective of experts. People are guided by using existing knowledge and attitudes, even though these might be linked to irrelevant concepts. They try to act rationally in a situation where they do not have the appropriate knowledge to do so. To be more precise, non-experts use less extensive schemata to process the information. The important differences between new and familiar concepts can be so subtle that they are not noticed or comprehended; and, even if people notice errors or imperfections in their logic, they still have no alternative.

For invisible technologies in particular, a name can have a severe impact on the interpretation of what it entails and the development of attitudes. Realizing this has several consequences for the development of a proper name. To avoid unnecessary controversies, it is beneficial for experts to anticipate the way non-experts give meaning when confronted with technical names. First, it is important to know that non-experts try to form understanding through internal transfer of knowledge and attitudes. When people are confronted with a new technology, the knowledge and attitudes that they use to assess it will probably come from a single familiar category or example. Experts names should try to guide the consumer to a particular source that holds valuable information. This guidance can be achieved by selecting a name that has value for the public.

Second, it is important to realize that what a name means for experts may not be the same as its meaning for the public. For example, the word gen might be understood completely differently because, in public debates, it is often linked to genetic manipulation. The real scientific meaning might therefore not exist in the minds of many non-experts. From a cognitive perspective, a name might activate unexpected schemata. In order to prevent instant misunderstanding, it is important to test whether or not the name actually activates information that is meaningful for understanding the concept or the main attribute.

When education does take place, people will pay more attention to information that is in line with expectations based on the activated schemata. In a study on the effects of providing information, Scholderer and Frewer (2003) found that providing information about biotechnology did not lead to attitude change, merely to the activation of pre-existing attitudes. The pre-existing attitudes form a judgement without knowledge, or a prejudiced judgement. In a way, people are prejudiced in favour of information that confirms activated beliefs. Activating the proper beliefs by a well-chosen name might enhance the success of an education programme.

A complicating aspect unmentioned so far is that categories and their association with names are not necessarily the same between cultures or stable over time. In some cases, it might be wise to select different names for different languages or cultures. This does not take away the fact that well-chosen names can provide an important advantage. It does mean, however, that deliberate attention to the context can enhance the benefits of selecting a name. In some cases such attention might even be crucial.

Overall, in order to reach the public, it is important not to try to achieve perfect understanding, but rather choose to achieve a good enough understanding. Instead of trying to enforce their own complex schemata on members of the public, scientists should focus on a category that is useful. Many technological names emphasize the relation of a technology to other technologies. The name is given with the extensive organization of other technologies in mind, and the name has meaning within this structure. Without this extensive schema, the name has no meaning. Trying to completely explicate the schema to a non-expert is probably a lost cause.

In the current article, we explored the way people without knowledge give meaning to genomics, with the gen part of the name being the central concept. We believe that the principle can be applied to other fields where people do not have the correct knowledge to differentiate between concepts, and where scientific names might have a different meaning in daily life. For example, nuclear is more associated with radiation than with atom cores, synthetic biology might cause people to believe that this is related to synthetic materials, and the extent to which people can tell the difference between harmful nanoparticles and the broader field of nanotechnology is highly questionable. In situations like these, negative attitudes might appear for invalid reasons and harm the technology purely due to misunderstanding. Wrong interpretations can cause unnecessary confusion and even cloud ethical debates.

It should be noted that we are in no way trying to argue that people in general are ignorant and that their opinions should not be respected, or that providing education is necessarily a bad thing. We do say that it is impossible to be educated and knowledgeable on everything. It is therefore, in our opinion, neither fair nor realistic to expect people to know everything, and not to accept public opinions based on imperfections of understanding. We have to accept that people are forced to use inferences and that, when education does take place, the development of knowledge can be influenced by the inferences made. When new technologies are being introduced, it is necessary to keep in mind everyone’s cognitive limitations. Instead of using a lack of knowledge against the public, experts should try to prevent incorrect understandings and try to guide people to meaningful knowledge. We want to stress that we do not suggest that experts should just pick a nicer sounding name. The suggestion that experts should investigate what kind of name creates meaningful inferences is meant to enhance understanding and prevent confusion.

It should also be noted that, of course, not all objections towards new technologies are the result of misunderstandings. It is true that it was expected by experts that due to genomics sharing key features of traditional breeding, it would be evaluated more favourably in comparison to GM (Hall 2010). It is also true that, as stated previously, GM and genomics share features. The current article is, however, based on observations by experts that people confuse technologies due to an (common) absence of knowledge (Nap et al. 2002; van Dam and de Vriend 2002), rather than not accept them. In the case of genomics, the technology that can have far-fetching consequences and people that have related knowledge can have well-reasoned arguments. These arguments and the people that have them do deserve attention. We do not wish to argue that genomics is –or should be- regarded as safe, acceptable or ethical. The problem we are addressing is the fact that people automatically assume that genomics is GM., which is neither correct nor a normative issue. Our interest in ignorance is in no way support for dismissing genuine concerns as resulting from a lack of knowledge.

In clouded debates, it is especially easy to see that there is a logic behind the idea that the public need to be educated, even though this idea is often disregarded due to the false belief that education equals persuasion. It should be noted that the idea of educating the public has not been developed only from the observation that people disagree; another important part is the observation that people, on occasion, do have misconceptions. The introduction of new technologies in society can definitely be harmed by a lack of understanding. Education could possible reverse this. Unfortunately, it would require too much education, on too many occasions and in unsuitable situations. Instead of trying to reach the public with external information, experts should try to utilize existing internal knowledge of members of the public. It is our advice to select names through a process of co-creation, in which not only public associations can be explored, but that give the opportunity to select associations which are deemed important by the public. To increase understanding, the right name can be a powerful tool.
